# Disparities in economic burden for children with leukemia insured by resident basic medical insurance: evidence from real-world data 2015–2019 in Guangdong, China

**DOI:** 10.1186/s12913-022-07564-8

**Published:** 2022-02-19

**Authors:** Chunwang Zhan, Zhiming Wu, Lihua Yang, Lihua Yu, Jie Deng, Kiuco Luk, Chongyang Duan, Luwen Zhang

**Affiliations:** 1 School of Health Management, Southern Medical University, 1023 Shatai Road, Baiyun District, Guangzhou, 510000 China; 2 Department of Healthcare Insurance, Nanfang Hospital, Southern Medical University, Guangzhou, China; 3 Department of Pediatric Hematology, Zhujiang Hospital, Southern Medical University, Guangzhou, China; 4 Department of Health Economic ManagementZhujiang Hospital, Southern Medical University, Guangzhou, China; 5 Department of Biostatistics, School of Public Health, Southern Medical University, 1023 Shatai Road, Baiyun District, Guangzhou, 510000 China

**Keywords:** Economic burden, Cost, Resident basic medical insurance (RBMI), Pediatric leukemia, Acute lymphoblastic leukemia (ALL), Acute myeloid leukemia (AML)

## Abstract

**Background:**

Pediatric leukemia is the most prevalent childhood cancer in China and incurs heavy economic burden to patients without sufficient insurance protection. Although all Chinese children are obliged to enroll in the national insurance scheme, “Resident Basic Medical Insurance (RBMI)”, the protection may vary among patient subgroups. This study is designed to measure the disparities in economic burden for patients with leukemia under RBMI protection and explore the influencing factors.

**Methods:**

The included patients were aged ≤ 15 and diagnosed with acute lymphoblastic leukemia (ALL) or acute myeloid leukemia (AML, with/without transplantation). They all completed treatment course consecutively in Nanfang Hospital and Zhujiang Hospital from Jan.1, 2015, to Dec.30, 2019, in Guangzhou, China. Their inpatient treatment and insurance settlement data were drawn from the Hospital Information System (HIS) and Insurance Settlement System (ISS). A total of 765 consecutive patients and 14,477 inpatient medical records were included and analyzed. Their insurance status (6 subtypes), economic burden [total cost, out-of-pocket cost (OOP), reimbursement, reimbursement rate (RR)], and cost structures (operation/procedure, blood products, drug, simple treatment) were calculated respectively. Non-normally distributed costs were reported as the median and interquartile range (IQR). Wilcoxon test was used for univariate tests and generalized linear model with log link was used to explore the influencing factors.

**Results:**

The insured patients who were treated in the location of insurance with instant reimbursement reported the highest total cost and reimbursement, while those who seek medical care cross-province with no instant reimbursement reported the lowest total cost and highest OOP payment. In terms of annual change, the total cost of children with leukemia decreased from 2015–2019 with stably increasing reimbursement rate. Blood products and drugs were the major components of total cost, but they decreased annually. Patients who received transplantation and treated across provinces were with a higher economic burden.

**Conclusion:**

The economic burden for children with leukemia decreased overtime under the protection of RBMI, but disparities exist among subtypes. The payer-provider contract on instant reimbursement and drug cost control are effective measures for insurance administrators to curb the economic burdens of pediatric leukemia treatment.

**Supplementary Information:**

The online version contains supplementary material available at 10.1186/s12913-022-07564-8.

## Background

Pediatric leukemia, or childhood leukemia, is one of the most prevalent hematological malignancies among childhood cancer [1]. The two major types, acute lymphoblastic leukemia (ALL) and acute myeloid leukemia (AML), take up 75% and 15%-20% of all cases [2]. With the advanced improvement in the clinical management of childhood leukemia, ALL and AML have achieved excellent prognoses by following standard treatment plans [3]. The treatment protocols are quality guaranteed and are strictly followed in China, and the both hospitals in our study followed “GD-2008-ALL protocol” [4] for ALL treatment and “C-HUANAN-AML15 protocol” [5] for AML treatment.

In addition to the quality of treatment, the affordability for treatment is of equal importance for patients with pediatric leukemia [6]. The complete treatment course of AML and ALL may take up to 3–5 years. The total treatment cost ranges from Chinese yuan (CNY) 300,000–500,000, and in cases with bone marrow transplantation the cost may exceed CNY 1 million in China [7]. Zeidan etc. compared the total cost and cost structure of AML treatment in the UK and the US. The average costs were £59,426 and $324,502 for intensive chemotherapy, £112,545 and $352,682 for allogeneic stem cell transplantation in the UK and the US, respectively [8]. The treatment of ALL and AML requires chemotherapy, anti-infection treatment, and immune support, which require patients to accept consecutive and consistent treatment from a stable and regular medical provider with a well-trained physician team and high-quality medical facilities [9]. The lack of sufficient insurance protection could easily incur heavy burdens for the patients and their families during the prolonged and expensive treatment process.

Insurance protection for children with leukemia is always a serious concern on government administration and health equity research. The international study of childhood leukemia shows that insurance coverage, equity, and capacity are the major research areas [10]. Researchers in high-income countries started investigating patient economic burdens as early as the1980s [11] and many initiatives were made to call for full access to care [12, 13]. In contrast, research in China started late and was generally underestimated by researchers on the economic burden. Wang etc. analyzed the causes, refusal, and abandonment of treatment in ALL from 1997–2007. They found that medical insurance and systemic health education are vital for families with ALL patients [14]. Zhou etc. examined administrative and clinical data of Soochow University Hospitals from 2002 to 2012. They found that two policies (providing funding for the catastrophic diseases and the New Rural Cooperative Medical System) initiated in 2005 and 2011 have significantly reduced the abandonment of ALL treatment in Jiangsu Province [15].

According to the World Health Organization (WHO) framework of health insurance coverage, there are three dimensions to measure health insurance protection: breadth—who is covered, depth—what is covered, and height—what proportion of the cost is covered [16]. Universal insurance coverage has been provided to children under 18 by RBMI in China since 2009. However, RBMI is pooled at prefecture city level, the reimbursement policies vary across cities. When cancer patients must seek treatments outside locations of insurance, they may face extra obstacles in getting reimbursement, including decreasing reimbursement rate (RR), narrowed reimbursement list, ineligible for instant reimbursement, etc., which may significantly increase the direct and indirect economic burdens for them [17].

The health insurance policies for pediatric leukemia have been improving positively since 2009. We made a thorough analysis of the progress of medical insurance policies for pediatric leukemia from 2009–2020. The universal RBMI could be divided into accurate subtypes by location of insurance, and payer-provider contracted reimbursement requirements. The comparison was made chronologically (from 2009–2020) and cross-regionally (in/out Guangdong Province) (Appendix 1). From 2009 to 2011, RBMI only covered inpatient costs for a limited disease list. After 2012, pediatric leukemia was entitled extra reimbursement quota from a supplementary plan embedded in RBMI named “Catastrophic Medical Insurance”(CMI), which offers CNY 300,000 annual entitlement, but only for children aged ≤ 15 [18]. The protection of RBMI kept increasing across the nation since then—the coverage rate increased, the reimbursement lists expanded, the reimbursement rates escalated, and low deductibles were adopted. These improvements were especially progressive after 2012 when pediatric leukemia was entitled as a special disease receiving extra reimbursement package from the national essential insurance plans [19]. Meanwhile, the patients’ insurance claim and reimbursement process have been simplified by pre-payment reimbursement contract between payers and providers. Large hospitals or cancer treatment centers are encouraged to sign direct reimbursement contracts with payers (Health Insurance Bureaus at prefecture-level) and submit electronic insurance claims for patients to payers, which allow patients to receive instant reimbursement when discharged. Since RBMI is pooled and administrated at the prefecture city level, the insurance protection may vary at the location of insurance, that is, the prefecture city where the patient enrolled in RBMI. Despite the complex negotiation and inconsistent speed of contracting, an increasing number of patients can receive instant reimbursement when discharged thanks to this reform.

This study explored the changing economic burdens and insurance protection for children with leukemia who received care in South China’s Guangdong Province from 2015 to 2019 using real-world data drawn from two regionally representative hospitals in Guangzhou, China. The data, including hospital treatment records, discharge settlement, and insurance settlements data, provided evidence of changes in economic burdens over time and insurance location.

## Methods

### Study settings

Guangzhou city (GZ), as the capital of Guangdong Province (GD), has a concentration of high-quality medical resources not only in Guangdong province but also in South China. Data used in this research were real-world data drawn from the two largest affiliated hospitals of Southern Medical Universities: Nanfang Hospital and Zhujiang Hospital. Both hospitals were nationally reputable teaching hospitals and medical research centers. The Department of Hematology and Pediatric Centers in the two hospitals take care of patients from both Guangdong province and across China. In our study, 79.7% of patients were from Guangdong and 20.3% were from provinces in South China, which made the study samples of good representativeness. Patients in two hospitals followed the same clinical guideline. The two hospitals followed the same clinical guidelines: the treatment of ALL followed “GD-2008-ALL protocol” [4] and AML treatment “C-HUANAN-AML15 protocol” [5].

### Data source

Data were drawn from the two hospitals’ administrative records from the year 2015 to 2019. Patients’ demographic information and treatment records were sourced from the hospital information system (HIS), while the costs and insurance reimbursement information were drawn from the health insurance discharge settlement system (DSS). Data analysis received the formal contracted approvals with strict limitation items from the Department of Administration of Southern Medical University, Zhujiang Hospital, and Nanfang Hospital. Data were de-identified and for academic use only. The study included patients who were diagnosed with ALL and AML (ICD C91.000, C92.000, and the subtypes) who received complete treatment course in the two hospitals from Jan.1, 2015, to Dec. 30, 2019. We only included patients aged ≤ 15 years due to the age restriction items in RBMI [18].

A total of 490 patients from Nanfang Hospital and 310 patients from Zhujiang Hospital were included at the first stage. One physician and two nurses who were familiar with their patients’ treatments from each study department worked together and completed the first screening. We excluded the patients who did not receive the complete treatment course in the two study hospitals. The complete treatment course of leukemia is characterized as follows—once the induction process of intensive treatment started, the patients would follow the treatment from the same physician team, and only in very rare cases did they transfer hospitals. Finally, a total of 765 consecutive patients and their 14,477 inpatient treatment medical records were included in this study.

### Disease types

Two types of diseases, ALL and AML (ICD C91.000, C92.000, and the subtypes), were included in the analysis. AML and ALL involve different hematopoietic dysfunction. AML impairs the production of myeloblasts, red blood cells, and platelets, whereas ALL mainly affects the production of lymphocytes. Because only the AML patients who meet the treatment criteria could receive transplantation, and the total cost of transplantation was generally 1–2 times more than AML without transplantation, we divided all patients into three disease types: ALL—patients with ALL only, AML—patients with AML but without transplantation, and Transplantation—patients with AML and received transplantation.

### Variables and coding

Two determinants were considered in categorizing the insurance types— the location of insurance, and the eligibility for the instant reimbursement at the time of discharge settlement. The location of insurance, due to the enrollment policies of RBMI in China, is generally decided by the Hukou status, namely the city the patient registered their household. The eligibility for instant reimbursement was decided by the contract between the medical insurance payers and medical service providers. The insured patients who received care in their location of insurance are generally entitled to instant reimbursement when discharged. However, for those who seek medical care out of the location of insurance, they may lose the eligibility for instant reimbursement and must prepay medical bills OOP and return to the location of insurance for the reimbursement. For patients with catastrophic diseases like leukemia, the postponed refund may increase patient’s economic burden and increase the risk of giving up treatment items.

A total of six insurance subtypes were categorized accordingly: GZ-RBMI, GD-contracted, GD-uncontracted, Other-contracted, Other-uncontracted, and OOP.

GZ-RBMI refers to the patients who had Guangzhou hukou and were covered by Guangzhou RBMI. They generally enjoyed instant reimbursement and the highest RR.

GD-contracted referred to patients who were enrolled in RBMI in the other cities in Guangdong Province, covered by RBMI, and eligible for instant reimbursement according to the payer-provider contract.

GD-uncontracted referred to patients who were enrolled in RBMI in the other cities in GD and covered by GD RBMI, but they were not eligible for instant reimbursement at the time of discharge. These patients had to pay for bills OOP and get reimbursed after returning to their location of insurance.

Other-contracted referred to the patients who were enrolled in RBMI outside GD by RBMI but eligible for instant reimbursement.

Other-uncontracted referred to the patients who were enrolled in RBMI outside GD province by RBMI but ineligible for instant reimbursement. They also had to pay for bills OOP and get reimbursed after returning to their location of insurance.

The OOP group referred to those who had no insurance records in HIS files and paid all bills OOP in ISS when discharged.

The economic burden of patients was described as OOP, reimbursement, and total cost, which referred to the out-of-pocket costs, reimbursed costs, and total costs paid by patients for the complete treatment course. The reimbursement rate (RR) was defined as the percentage of reimbursement divided by total cost. The types of cost here referred to the added cost during the complete treatment course.

The cost structure was described by four types: operation/procedure cost, simple treatment cost, blood products cost, and drug cost following the ISS record structure.

The treatment duration refers to the average number of days required to complete the treatment process. The average inpatient days equaled total inpatient days divided by times of hospitalization. Three age groups were divided, 0–5, 6–10, and 11–15. The regions were categorized according to where the patients came from: the developed Pear-River Delta cities in Guangdong Province (GD-PRD), the less developed east, west, and north wings of Guangdong Province (GD-Other), and the other provinces outside GD (Outside GD).

### Statistics

The statistical description was made to report the distribution of all variables. The cost was described as non-normal distributed and reported as the median and interquartile range (IQR). Wilcoxon test was used to make the univariate analysis of cost data. Generalized linear model with log link was also used to analyze the influencing factors of economic burden and cost structure. The cost was logarithmically transformed to the normal distribution and then the coefficients are exponentially transformed accordingly. All analyses were conducted using SAS version 9.3 (SAS Institute, Inc). Two-tailed *p* < 0.05 was considered statistically significant.

## Results

### Demographic characteristics and cost information

A total of 765 patients who received complete treatment rounds in the two hospitals were included in this study, 261(34.1%) patients were from developed Pear-River Delta area in Guangdong province, 349(45.6%) from less developed north, east and west wings of Guangdong, and 20.3% from the other provinces in South China except Guangdong. Four hundred and sixty-nine (61.3%) patients were treated in Nanfang Hospital and 296(38.7%) in Zhujiang Hospital. Among all patients, 596 (77.9%) were diagnosed with ALL, 111(14.5%) with AML, and 58 (7.6%) received stem cell transplantation. As for the insurance types, 75(9.8%) of them were covered by GZ-RBMI, 412 (53.9%) by GD-contracted, 60 (7.8%) by GD-uncontracted, 38 (5.0%) by Other-contracted plans, and 46 (6.0%) by Other-uncontracted plans. Additionally, a total of 134 (17.5%) patients reported complete OOP payments during the treatment process (Table 1).

**Table 1:** Socio-economic characteristics, costs distributions, and treatment duration of children with leukemia

Variables	Code	Freq. (%)	Variables	Code	Value
*Demographic information*			*Total Costs & reimbursement (CNY/%)*		
**Leukemia Type**	ALL	596(77.9%)	**Total Cost**	Median	244,250.1
	AML	111(14.5%)		IQR	156,330.8–357,847.4
	Transplantation	58(7.6%)	**Total OOP**	Median	182,860.2
**Hospitals**	Nanfang H	469(61.3%)		IQR	112,324.1–266,849.7
	Zhujiang H	296(38.7%)	**Total Reimbursement**	Median	47,575.45
**Insurance Type**	GZ-RBMI	75(9.8%)		IQR	0.00–115,375.0
	GD-contracted	412(53.9%)	**RR**	Median	24.16
	GD-uncontracted	60(7.8%)		IQR	0.00–39.74
	Other-contracted	38(5.0%)			
	Other-uncontracted	46(6.0%)	*Cost structures (CNY)*		
	OOP	134(17.5%)	**Operation/Procedure**	Median	481.59
**Year**	2015	212(27.7%)		IQR	286.00–867.59
	2016	147(19.2%)	**Simple Treatment**	Median	0.00
	2017	170(22.2%)		IQR	0.00–0.00
	2018	144(18.8%)	**Blood Products**	Median	12,095.00
	2019	92(12.0%)		IQR	5760.00–24,125.00
**Sex**	Male	482(63.0%)	**Drug**	Median	91,486.07
	Female	283(37.0%)		IQR	44,721.65–152,689.1
**Age**	0–5	419(54.8%)			
	6–10	190(24.8%)	*Treatment Duration (Days)*		
	11–15	156(20.4%)	**Total Treatment Duration**	Median	526.00
**Region**	GD-PRD	261(34.1%)		IQR	319.00–764.50
	GD-Other	349(45.6%)			
	Outside GD	155(20.3%)	**Total Inpatient Days**	Median	164.00
**Hukou**	Urban	347(45.4%)		IQR	126.00–213.50
	Rural	418(54.6%)			

Transplantation, the AML patients who received transplantation. GD-PRD, the Pear-River Delta cities in Guangdong Province. GD-Other, the other cities in Guangdong outside Pear-River Delta regions

*RR* reimbursement rate, *CNY* Chinese Yuan, *IQR* interquartile range, *OOP* out-of-pocket payment, *ALL* acute lymphoblastic leukemia, *AML* acute myeloid leukemia

The median of the complete treatment process was 526 (319.0- 764.5) days, and the median of inpatient days was 164 (126.0–213.5) days. The total median total, total OOP cost and total reimbursement per patient was CNY244,250.1(156,330.8–357,847.4), CNY182,860.2 (112,324.1–266,849.7), and CNY47,575.45 (0.00–115,375.0) respectively. The median reimbursement rate (RR) was 24.16% (0.00–39.74%). As for the cost structures, the drug cost per patient was the highest at CNY 91,486.07(44,721.65–152,689.1), followed by the cost of blood products CNY12,095.00 (5,760.00–24,125.00), and operation/procedure cost CNY481.59 (286.00–867.59) (Table 1).

### Economic burdens for subgroups

Table 2 reported the cost burdens for subgroups and the insurance protection enjoyed by each group. The average economic burdens were generally alleviated by later years. The median annual total cost decreased from CNY 103,381.9(60, 223.97–188,093.9) in 2015 to CNY55,551.60(11,747.07–152,260.7) in 2019 (P < 0.01) with annually escalating RR, indicating the effect of payment controls and improved protection. As for the disease types, the patients received transplantation reported higher total cost CNY535,881.1(424,825.7–783,175.1) than patients with AML CNY301,265.2(218,178.0–415,918.3) and ALL CNY 218,084.7(141,338.0–312,535.4) (*P* < 0.01), but they also received the highest RR (29.18%) than AML (26.97%) and ALL (23.77%) patients (*P* < 0.01). As for the insurance subtypes, patients with GZ-RBMI reported the highest total cost CNY 276,519.6 (164,781.8–424,408.5), lowest OOP cost CNY172,992(107,911.7–294,540.2), and highest RR 36.02% (29.56%-43.01%). Patients in the GD-contracted group and Other-contracted group were reported slightly lower total cost [CNY275,138.1(178,242.0–413,903.0), CNY273699.3(191,074.4–348,430.8)], but the instant reimbursement was much higher for patients receiving care within province than outside province (35.16% vs 23.08%, *P* < 0.001).

**Table 2:** The univariate analysis for total cost, OOP, reimbursement, and reimbursement rate of children with leukemia

Variable	Code	Total Cost (CNY)		OOP (CNY)		Reimbursement (CNY)		RR (%)	
		**Median**	**IQR**	**Median**	**IQR**	**Median**	**IQR**	**Median**	**IQR**
**Disease Type**	**ALL**	218,084.7	141,338.0–312,535.4	165,601.3	101,462.5–243,743.3	41,378.19	0.00–100,648.6	23.77	0.00–37.51
	**AML**	301,265.2	218,178.0–415,918.3	224,771.8	153,500.4–296,861.8	87,791.30	0.00–165,320.2	26.97	0.00–43.59
	**Transplantation**	535,881.1	424,825.7–783,175.1	398,933.7	237,443.5–536,682.6	162,134.7	0.00–371,971.7	29.18	0.00–56.76
	**P value**	***		***		***		***	
**Insurance subtype**	**Guangzhou RBMI**	276,519.6	164,781.8–424,408.5	172,992	107,911.7–294,540.2	95,001.67	46,119.94–144,048.6	36.02	29.56–43.01
	**GD-contracted**	275,138.1	178,242.0–413,903.0	180,306.6	108,787.0–265,812.9	91,881.23	44,683.79–161,344.2	35.16	23.51–46.50
	**GD-uncontracted**	165,241.0	78,184.73–224,379.1	165,241.0	78,184.73–224,379.1	0	0.00–0.00	0	0.00–0.00
	**Other-contracted**	273,699.3	191,074.4–348,430.8	198,347.4	159,018.7–253,269.8	64,180.55	36,478.73–102,633.9	23.08	13.16–34.32
	**Other-uncontracted**	251,090.2	164,543.3–310,643.8	251,090.2	164,543.3–310,643.8	0	0.00–0.00	0	0.00–0.00
	**OOP**	177,433.7	107,366.9–273,631.8	177,433.7	107,366.9–273,631.8	0	0.00–0.00	0	0.00–0.00
	**P value**	***		***		***		***	
**Year**	**2015**	103,381.9	60,223.97–188,093.9	82,487.89	37,817.86–144,893.0	0.00	0.00–38,610.20	0.00	0.00–36.44
	**2016**	90,205.78	27,515.77–153,661.0	59,752.63	21,198.28–132,200.6	0.00	0.00–31,896.13	0.00	0.00–37.98
	**2017**	85,075.54	15,820.08–174,135.1	54,219.21	13,373.37–136,003.3	1840.77	0.00–48,230.88	14.69	0.00–37.03
	**2018**	82,765.10	14,040.39–202,800.7	61,490.79	10,386.24–148,066.5	5274.70	0.00–60,622.59	24.98	0.00–39.17
	**2019**	55,551.60	11,747.07–152,260.7	40,780.51	8834.24–114,992.2	6311.06	0.00–40,160.43	24.89	0.00–36.68
	**P value**	**		***		***		***	
**Age**	**0–5**	220,654.9	145,597.8–303,492.5	171,986.8	106,132.7–246,369.6	38,820.03	0.00–94,410.15	22.73	0.00–36.99
	**6–10**	244,457.1	156,938.0–388,577.1	179,391.5	115,559.3–273,534.0	52,122.70	0.00–116,160.6	19.87	0.00–42.12
	**11–15**	333,470.6	198,986.3–536,133.8	227,305.0	144,895.5–318,728.3	94,356.12	0.00–201,191.5	29.49	0.00–48.85
	**P-value**	***		***		***		*	

Transplantation, the AML patients who received transplantation

*CNY* Chinese Yuan, *OOP* out-of-pocket payment, *ALL* acute lymphoblastic leukemia, *AML* acute myeloid leukemia

^*^ *p* < 0.05, ^**^ *p* < 0.01, ^***^ *p* < 0.001, significance test by Wilcoxon Test

### Cost structures for subgroups

Table 3 reported the costs for the major service item combinations. Drug and blood transfusion costs took the largest quota of the total costs. Patients with AML and transplantation reported 3-times more blood costs and 1–2 times more drug cost than ALL patients (*P* < 0.001). The blood and drug costs showed decreasing trend from 2015–2019. The blood products cost decreased by 40% in 2019 compared to 2015 [CNY1,290(0–8,090) vs. CNY6090(862.50–14,103.75), *P* < 0.00] and drug cost by 20% [CNY23,410.3(3,768.02–65,276.35) vs. 36,810.68(12,827.45–79,488.75), *P* < 0.001], indicating the effectiveness of cost control measures released by insurance administration sectors. Meanwhile, the contracted insurance plans that offered patients instant reimbursement reported significantly higher costs and drug costs than the uncontracted plans.

**Table 3:** The univariate analysis of cost structures for children with leukemia (CNY)

Variable	Code	Operation and Procedure (CNY)		Simple Treatment (CNY)		Blood Products (CNY)		Drugs (CNY)	
		**Median**	**IQR**	**Median**	**IQR**	**Median**	**IQR**	**Median**	**IQR**
**Disease Type**	**ALL**	533.34	321.75–886.56	0	0.00–0.00	9582.5	4108.75–17,950.00	78,281.97	35,146.59–130,500.9
	**AML**	275	178.75–445.84	0	0.00–0.00	28,390	16,980.00–42,915.00	128,808.2	81,721.10–209,854.2
	**Transplant**	708.63	344.56–1387.78	0	0.00–81.26	30,615	17,708.75–52,890.00	202,692.4	128,886.9–312,764.2
	**P value**	***		*		***		***	
**Insurance Types**	**Guangzhou RBMI**	575.00	286.00–960.00	0	0.00–26.65	13,755.00	5620.00–25,490.00	103,672.3	57,349.20–176,983.1
	**GD-contracted**	377.93	250.19–791.63	0	0.00–0.00	11,845.00	3231.25–18,912.50	72,127.95	23,839.42–120,908.0
	**GD-uncontracted**	528.13	321.75–894.69	0	0.00–6.00	12,712.50	6568.75–25,261.00	99,375.42	51,600.11–166,577.5
	**Other-contracted**	362.75	249.31–532.96	0	0.00–0.00	19,570.00	11,745.00–35,356.25	128,931.0	80,694.81–192,108.7
	**Other-uncontracted**	411.13	282.38–737.01	0	0.00–18.95	14,615.00	8388.25–22,543.75	105,486.9	62,317.89–126,769.1
	**OOP**	484.73	241.13–821.25	0	0.00–0.00	8377.50	3510.00–17,041.25	55,639.61	22,698.34–101,933.8
	**P value**	*				***		***	
**Year**	**2015**	200	108.25–350.00	0	0.00–0.00	6090	862.50–14,103.75	36,810.68	12,827.45–79,488.75
	**2016**	225	100.00–450.00	0	0.00–0.00	3820	0.00–10,900.00	29,735.27	8392.77–79,249.36
	**2017**	185.75	71.50–384.13	0	0.00–0.00	3300	0.00–13,080.00	18,019.46	3618.26–62,113.58
	**2018**	178.75	71.50–432.25	0	0.00–71.50	2410	0.00–11,047.50	20,360.5	2211.02–74,777.05
	**2019**	143	35.75–237.90	0	0.00–0.00	1290	0.00–8090.00	23,410.3	3768.02–65,276.35
	**P value**	***		***		***		***	
**Age**	**0–5**	500.75	286.00–930.50	0	0.00–0.00	11,110	4710.00–18,887.50	81,721.1	34,200.97–129,826.9
	**6–10**	450.6	275.00–755.00	0	0.00–6.00	13,755	6780.00–29,885.00	102,340.2	55,163.28–172,353.3
	**11–15**	475	314.50–796.95	0	0.00–35.75	17,255	8500.00–36,135.00	128,519	59,518.20–209,854.2
	**P value**			*		***		***	

Transplantation, the AML patients who received transplantation

*CNY* Chinese Yuan, *OOP* out-of-pocket payment, *ALL* acute lymphoblastic leukemia, *AML* acute myeloid leukemia

^*^ *p* < 0.05, ^**^ *p* < 0.01, ^***^ *p* < 0.001, significance test by Wilcoxon Test

### Influencing factors

Table 4 reported the influencing factors on total cost, reimbursement cost, drug cost, and blood product cost. Patients covered by GZ-RBMI and GD-contracted had total costs 1.6 and 1.5 times higher than that of OOP patients with all else equal, which indicated the protective effect of provider-payer contracted instant reimbursement on the medical service affordability and higher possibility of fulfilling healthcare needs. The total cost and reimbursement from 2016–2019 reported similar fluctuation of ratios compared with the cost in 2015 (1.3–1.6 times, *P* < 0.05), implying the robust cost change and protective effect of the reimbursement mechanism. It confirmed the hypothesis of two dimensions of subtypes of insurance—location of insurance and contraction of reimbursement—on cost burden. As for disease types, AML and transplantation patients reported 1.492 and 3.456 times higher than ALL patients in total cost (*P* < 0.000) and 1.246 and 2.829 times (*P* < 0.005) in reimbursement, which indicate the cost burden was disparate among diseases.

**Table 4:** Multiple regression of factors influencing the total cost, reimbursement, drug cost and blood cost for children with leukemia

Variable	Model 1: Total Cost				Model 2: Reimbursement				MODEL 3: Drug Cost				MODEL 4: Blood Products Cost			
	**Beta**	**Exp(Beta)**	**S.E.**	**P-value**	**Beta**	**Exp(Beta)**	**S.E.**	**P-value**	**Beta**	**Exp(Beta)**	**S.E.**	**P-value**	**Beta**	**Exp(Beta)**	**S.E.**	**P-value**
**Disease type** (Ref.ALL)																
AML	0.400	1.492	0.080	0.000	0.220	1.246	0.100	0.022	0.670	1.954	0.110	0.000	1.000	2.718	0.100	0.000
Transplantation	1.240	3.456	0.120	0.000	1.040	2.829	0.140	0.000	1.380	3.975	0.150	0.000	1.430	4.179	0.140	0.000
**Insurance type** (Ref. OOP)																
GZ-UBMI	0.480	1.616	0.160	0.003	0.090	1.094	0.170	0.607	0.550	1.733	0.290	0.057	0.310	1.363	0.260	0.246
GD-uncontracted	-0.640	0.527	0.160	0.000	-0.630	0.533	0.160	0.000	0.880	2.411	0.190	0.000	0.180	1.197	0.180	0.305
GD-contracted	0.410	1.507	0.120	0.001	-0.060	0.942	0.120	0.611	0.810	2.248	0.160	0.000	0.030	1.030	0.150	0.861
Other-uncontracted	-0.190	0.827	0.170	0.251	-0.290	0.748	0.170	0.087	0.640	1.896	0.300	0.031	0.360	1.433	0.270	0.185
Other-contracted	0.150	1.162	0.170	0.391	-0.220	0.803	0.170	0.203	0.340	1.405	0.210	0.102	0.260	1.297	0.200	0.183
**Year** (Ref. 2015)																
2016	0.510	1.665	0.090	0.000	0.490	1.632	0.090	0.000	0.620	1.859	0.120	0.000	0.110	1.116	0.100	0.282
2017	0.410	1.507	0.090	0.000	0.350	1.419	0.090	0.000	0.030	1.030	0.120	0.787	0.010	1.010	0.100	0.908
2018	0.490	1.632	0.090	0.000	0.470	1.600	0.100	0.000	0.340	1.405	0.120	0.005	-0.110	0.896	0.110	0.319
2019	0.270	1.310	0.120	0.022	0.220	1.246	0.120	0.067	0.100	1.105	0.150	0.526	-0.300	0.741	0.140	0.031
**Age** (Ref. 0–5)																
6–10	0.020	1.020	0.070	0.734	-0.010	0.990	0.070	0.904	0.120	1.127	0.090	0.215	0.280	1.323	0.080	0.001
11–15	0.270	1.310	0.080	0.001	0.200	1.221	0.080	0.012	0.380	1.462	0.100	0.000	0.480	1.616	0.090	0.000
**Region** (Ref. GD-DRP)																
GD-other	0.120	1.127	0.140	0.383	-0.140	0.869	0.150	0.344	0.100	1.105	0.180	0.570	-0.370	0.691	0.170	0.031
GD-outside	-0.070	0.932	0.080	0.410	-0.320	0.726	0.170	0.055	0.050	1.051	0.100	0.647	-0.060	0.942	0.090	0.495
**Hospital** (Ref. Nanfang)																
Zhujiang	0.620	1.859	0.080	0.000	0.740	2.096	0.080	0.000	1.010	2.746	0.100	0.000	0.970	2.638	0.090	0.000
**Hukou** (Ref. Urban)																
Rural	-0.010	0.990	0.060	0.847	0.010	1.010	0.070	0.920	0.010	1.010	0.080	0.863	0.100	1.105	0.070	0.200
**Sex** (Ref. Male)																
Female	0.020	1.020	0.060	0.785	0.010	1.010	0.060	0.910	0.030	1.030	0.080	0.732	-0.040	0.961	0.070	0.602

Beta, the regression coefficient of OLS model. Exp (Beta), coefficient transformed exponentially

*SE* standard error

As for the cost structures, patients with AML and transplantation reported 1.954 and 3.975 times higher drug cost and 2.718 and 4.179 times blood product cost compared with ALL patients (*P* < 0.000) controlling for other factors. Patients aged 11–15 years old reported 1.462 times higher drug cost and 1.616 times higher blood product cost compared with those aged 0–5. Additionally, patients from rural areas reported lower total costs but higher reimbursement than urban patients.

## Discussion

In the present study, we found decreasing total and OOP costs and increasing RR of RBMI for pediatric leukemia treatment from 2015 to 2019. Children with leukemia have constantly benefited from the universal insurance coverage achieved since 2009 health system reforms. The economic burdens were gradually alleviated over time. It has been estimated that a total of 13.7 million new cases of childhood cancer will emerge globally before 2050, and 11.1 million will die if no additional investments are made to improve access to healthcare services or childhood cancer treatment [20]. Of this total, 9.3 million children (84·1%) will be in low-income and lower-middle-income countries [21]. China’s achievements are adaptable to most middle- and low-income countries and it is worth exploring the reasons behind the achievements.

On one side, we have found a significant decrease in drug costs and blood costs for all diseases groups. This achievement may be attributed to a series of reforms after 2009. The payment method reform started in 2013 to harness the rapidly increasing inpatient costs should be the most significant contributor, where pre-payment replaced fee-for-service as the new method to regulate inpatient costs [22]. Among all reasons. the most influencing measures on cost decreased from 2015 to 2019, as far as we concerned, are government policies. Since 2015, all pilot cities of public hospital reform in China have allowed the zero-markup drug policy (ZMDP) [23] and implemented the policy of Separating Hospital Revenue from Drug Sales (SHRDS) [24]. Then RBMI limited the prescription of unnecessary supplement drugs, including Chinese patent medicine, vitamins, intravenous nutrition, etc. The Centralized Procurement policy implemented in Jan. 2019 has started a new era of drug price control. The state insurance administrator organizes centralized drug procurement and uses pilot programs to achieve significant reductions in drug prices to reduce transaction costs for enterprises and reduce the burden of drug costs for patients [25]. This policy has also guided medical institutions to standardize drug use and support public hospital reform. In late 2019, the State Organization of Centralized Drug Procurement and the Joint Procurement Office for the Use of Medicines has released the national centralized drug procurement documents in late 2019. Of these, 33 varieties were selected for the second national drug "centralized procurement" list, which covered drugs for major chronic diseases, as well as drugs for anti-tumor and rare diseases. This will further control and regulate cancer drug pricing [26].

Though it has been found that the total costs decreased and insurance protection improved in this research, there still exist gaps and issues to be solved. The average RR in this research ranged from 14 to 37%, which was much lower than the internationally defined level of protective insurance plans at 80% in the high-income countries [27, 28]. Sui etc. conducted a survey to understand the catastrophic health expenditures of families with pediatric leukemia patients. They found that the households covered by a single public health medical insurer were more likely to experience catastrophic health expenditure [29]. One explanation for this was the limited benefit package and low compensation rate. Another reason, according to interviews with hematologists, was the treatment of relapsing infection and complications [30]. The cost for these treatments is not included in benefits packages for pediatric leukemia [8]. The insurance administrators should notice this problem and collect more evidence from real-world data to explore the root of the problem. Clinical experts and hematologists should be invited to participate in designing reimbursement lists and insurance benefit packages, making sure the necessary drugs and treatment items and are included for reimbursement at a reasonable percentage in accordance with clinical protocols. Meanwhile, holistic supportive measures should be adopted and reimbursed by the insurance plans to prevent the development of infection and unnecessary complications [31]. For example, nutrition support and clinical exercise therapies have been proven to be cost-effective and should be supported by insurance plans [9, 32].

We also found that the protective effect of RBMI is significantly influenced by the off-site invitation: the contracted RBMI reported much higher RR and total costs than the uncontracted plans. Previous studies have demonstrated that immediate reimbursement would significantly influence the accessibility to care for children with catastrophic diseases in China [33, 34]. The payer’s willingness decides the instant reimbursement contract between payer and provider, and in this case, the insurance administrators at the prefecture-level [35]. It is also tightly related to the economic development of the places where patients are insured. Hong’s survey of clinical data of AML reported a negative association between the township size and treatment abandonment rate [36]. The patients from cities generally had the better economic capacity and enjoyed better insurance benefits. This regional disparity is worth noticing and should be corrected.

The younger patients reported much lower total costs than the older patients, but they were protected at lower RR compared to older patients. This positive linear trend indicated the age-related disparity in insurance protection. Clinical research has proven that younger patients always have lighter symptoms and better long-term health outcomes after treatment [37]. Therefore, policies should be made to adjust the benefit packages for infants and toddlers and distribute more resources to the children aged 0–10 to alleviate their economic burdens more efficiently [38]. Primary preventive measures should be taken to prevent pre-and postnatal events that are critical to leukemia development, which will need support from payers [39].

The patients who received transplantations reported the highest cost and RR in our study, demonstrating the effects of good insurance protection. The high cost of transplantation would be catastrophic for patients’ families without insurance protection [40]. The mental, emotional, and physical burdens to the patient and family who face bone marrow transplants are much higher than those receiving the general treatments process. In addition, bone marrow transplant faces many challenges such as hepatic venous obstruction disease, graft-versus-host disease, diffuse alveolar hemorrhage. These challenges increase the probability of implantation failure and severe infections. Even a successful transplant does not stop the future recurrence of the disease [41]. The essential public health insurance cannot be used as the only source of reimbursement, and the families should be conscious of supplementary commercial insurances to supplement their protection.

Another interesting result found in this study was the disparate total costs between the two affiliated hospitals. The direct cost of leukemia depends substantially on the treatment approaches the hospitals and their physician teams prefer. This finding indicates significant differences in treatment plan choice—even between two affiliated hospitals of the same medical university. To the standardized treatment of childhood leukemia, in August 2018, the National Health Commission issued *The Notice on the Management of Treatment and Management of Childhood Leukemia* to expand the standard treatment of childhood leukemia, which established a diagnosis and treatment network including 176 provincial-level designated hospitals and 374 prefectural-level designated hospitals. It strengthened the hierarchical diagnosis and treatment system and guidance and support system for childhood leukemia [42]. This network also allows children’s leukemia diagnosis and treatment institutions across the country to collaborate more closely. Five national-level Children’s Medical Centers (Beijing Children’s Hospital, Fudan University Pediatric Hospital, Shanghai Children’s Medical Center, Guangzhou Women’s and Children’s Medical Center, and Wuhan Children’s Hospital) lead the pediatric specialty alliance. These institutions guide subordinate hospitals through joint construction, practice guidance, and telemedicine, to improve the accessibility of high-quality medical services for children with leukemia in their proximity.

## Limitation

This study had a few limitations to consider. First, due to the current fragmented insurance system, we could not follow the reimbursement conditions for patients covered by GD-uncontracted and Other-uncontracted insurance providers. We will seek support from higher-level data sources from the provincial or even national Bureau of Health Insurance to solve this problem. Second, the costs data were not normally distributed, which constrained the choices of regression models. Third, the second-hand data drawn from the HIS and DSS systems covered a limited number of variables, which restricted this research design, and we will explore the remedial solutions by conducting patient interviews and investigations. Fourth, we did not further expand the study to the discussion of service items, disease stage, or health status in this research due to the limited article length, but we will further explore these influencing factors. Finally, we did not calculate the CPI discount in this paper, but we will consider the CPI effect in the following research.

## Conclusion

The economic burden for children with leukemia decreased overtime under the protection of RBMI, but disparities exist among subtypes. The cost burden varied among disease types, and patients who received transplantation had a heavier cost burden than AML and ALL patients. Instant reimbursement is an effective protective factor to remove the cost burdens in all disease types. The drug cost and blood products cost also reported an annual decrease from 2015–2019. The insurance administrators should elaborate on the payer-provider contract on instant reimbursement with more medical organizations to further alleviate the patient’s cost burdens. Meanwhile, they should proceed with the refined management of the medical insurance reimbursement catalog of pediatric leukemia to curb the escalating costs.

## Supplementary Information

**Additional file 1:** **Appendix 1. **The comparison of reimbursement policies in and out of Guangdong Province from 2009 to 2020.

## Data Availability

The data were acquired after receiving the formal, contracted approval of the department of administration of Southern Medical University, Zhujiang Hospital, and Nanfang Hospital under strict limitations. Data were de-identified before being handed to the research team, and the authors do not know of any information that can identify participants. The data are used only for academic purposes, and no one outside the team had the access to the data. The data are not publicly available due to the privacy restriction.

## Abbreviations

ALL: Acute lymphoblastic leukemia
AML: Acute myeloid leukemia
RBMI: Resident basic medical insurance
CNY: Chinese yuan
OOP: Out-of-pocket payment
GZ: Guangzhou city
GD: Guangdong Province
