# Supplementary material for: Disparities in economic burden for children with leukemia insured by resident basic medical insurance: evidence from real-world data 2015–2019 in Guangdong, China
Source: BMC Health Serv Res. 2022 Feb 19;22:229. doi: 10.1186/s12913-022-07564-8 (PMC8858506; doi:10.1186/s12913-022-07564-8)
Supplement: Supplementary file 1 — Additional file 1: Appendix 1. The comparison of reimbursement policies in and out of Guangdong Province from 2009 to 2020. [file 12913_2022_7564_MOESM1_ESM.docx]

**Appendix 1 The comparison of reimbursement policies in and out of Guangdong Province from 2009 to 2020.**

|  | **Reimbursement Policies in GD Province ^[[1]](#footnote-1)^** | | | **Reimbursement Policies out of GD Province** | |
| --- | --- | --- | --- | --- | --- |
| **Insurance Subtype** | **GZ-RBMI** | **GD-contracted** | **GD-uncontracted** | **Other-contracted** | **Other-uncontracted** |
| **Location of Insurance** | Guangzhou City | Cities in Pear-River Delta | Cities in the East, West and North Wings of GD | Outside Guangdong Province^#1^ | Outside Guangdong Province^#2^ |
| **Payer-provider Contract** | GZ-RBMI offer instant reimbursement and highest RR for the insured Guangzhou patients. | Prefectures contracted with the 2 hospitals; patients enjoy similar RR & list compared to patients treated in home city. | Some prefectures signed contract with 2 hospitals; patients enjoy lower RR & limited list compared to patients treated in home city. | Not contracted with 2 hospitals. | Not contracted with 2 hospitals. |
| **2009-2011** | Cover only inpatient costs. Treated as common diseases with no additional subsidies. Reimbursed 80% from primary care, 70% from secondary and 60% from tertiary hospital. Annual ceiling at CNY100,000. | | | Similar policy with higher annual ceiling at CNY 150k-200k. | Similar policy but lower reimbursement rate. |
| **2012-2014** | Pediatric leukemia was entitled secondary reimbursement from RBMI nationally. Reimbursement increased 5 percent point and annual ceiling improved to CNY100-150k average. | | | Similar to Guangdong. | Lower annual ceiling, and Cooperative medical scheme increased ceiling to 8 times average annual family income of peasants. |
| **2015-2016** | 18k enter plan B  annual ceiling 120k  average RR 70%  (CNY) | -- | -- | Deductible line to 50k: 50% reimbursed; over 50k:60%;  Annual ceiling 300k.  deductible line: 20k  (CNY) | Deducible line for Plan B 15k,  annual ceiling 300k.  Reimbursement.  15-50k, 50%  50-100k:55%；  over 100k 65%.  (CNY) |
| **2017-2018** | 2017  20k: enter plan b  20-50k, 50%,  50-100k, 60%,  >100k, 70%,  annual ceiling 300k  (CNY) | deductible 20k.  CNY20-50k, 60%,  CNY50-100k, 70%;  CNYOver 100k, 80%  Annual ceiling 300k (special drugs on list exempted).  (CNY) | 2017: deductible 10k. 10-30k, 55%, >30k, 70%; Annual ceiling 300k  (CNY) | Deductible line to 50k: 60% reimbursed; over 50k:70%;  Annual ceiling 300k.  deductible line: 20k  (CNY) | Deducible line for Plan B 15k, with annual ceiling 400k.  Reimbursement.  15-50k, 50%.  50-100k 60%；  over 100k: 70%.  (CNY) |
| **2019-2020** | Reimbursement:  18k to 50k, 60%,  >50k, 90%;  ceiling for insured for 1 year, 400k; ceiling for insured for 2 years in a row: 450k  (CNY) | Same with previous policy | Same with 2018 policy. | 2019: deductible line 30404,  Deductible line to 50k: 60% reimbursed; over 50k:70%;  Annual ceiling 300k.  Annual ceiling cancelled.  (CNY) | Same with 2018 policy |

**Note:** ^#1^, use Beijing City as example. ^#2^, use Henan Province as an example. RR, reimbursement rate. CNY, Chinese Yuan.

1. GZ represents Guangdong province. The east, west and north region of GD is less developed compared to Pearl River Delta Region. [↑](#footnote-ref-1)
